# Pain Resilience Therapy vs. Pain Neuroscience Education for Adults with Chronic Low Back Pain: Secondary Analysis of Data from a Randomized Controlled Trial of Risk and Resilience Factors

**DOI:** 10.3390/healthcare14131940

**Published:** 2026-07-01

**Authors:** Joe Tatta, Kerstin M. Palombaro, Janet R. Bezner, Rose M. Pignataro, Carey E. Rothschild

**Affiliations:** 1 Integrative Pain Science Institute, South Kent, CT 06785, USA; 2 Department of Physical Therapy, Arcadia University, Glenside, PA 19038, USA; 3 Institute for Physical Therapy Education, Widener University, Chester, PA 19013, USA; kpalombaro@widener.edu; 4 Department of Physical Therapy, Texas State University, Round Rock, TX 78665, USA; jb25@txstate.edu; 5 Department of Physical Therapy, Emory & Henry University, Marion, VA 24354, USA; rpignataro@emoryhenry.edu; 6 Program in Physical Therapy, University of Central Florida, Orlando, FL 32816, USA; carey.rothschild@ucf.edu

**Keywords:** Pain Resilience Therapy, Pain Neuroscience Education, chronic low back pain, psychological risk factors, pain resilience, OSPRO-YF, randomized controlled trial, secondary analysis

## Abstract

**Highlights:**

**What are the main findings?**

- Pain Resilience Therapy produced significantly greater improvements than PNE across 8 of 11 psychological outcomes, including pain catastrophizing, kinesiophobia, self-efficacy, and pain acceptance.
- Treatment response analysis demonstrated that participants in the PRT group were up to 6.5 times more likely to achieve an improvement than those in the PNE group.

**What are the implications of the main findings?**

- PRT simultaneously reduces psychological risk factors and enhances psychological resilience factors, supporting a dual-focus approach to pain care.
- PRT is feasible via telehealth and may be integrated into physical therapist-led pain management programs.

**Abstract:**

**Background:** Chronic low back pain (CLBP) is associated with psychological risk and resilience factors that drive pain, distress, and disability. Pain Resilience Therapy (PRT) is a novel strengths-based approach; however, its risk and resilience outcomes compared with recognized approaches remain unclear. **Objective:** To examine the effectiveness of PRT compared to Pain Neuroscience Education (PNE) on psychological risk and resilience factors in adults with CLBP. **Design:** Secondary data analysis from a randomized controlled trial. **Methods:** This secondary analysis included data from 60 adults with CLBP randomized to eight telehealth sessions of PRT or PNE delivered over four weeks. Psychological risk and resilience factors were assessed at baseline and at the 90-day follow-up using the OSPRO-YF, which estimates scores of 11 validated questionnaires across three domains: negative mood, negative/maladaptive coping, and positive affect/coping. Between-group differences were examined using ANCOVA with baseline adjustment. Treatment response was quantified using relative risk, absolute risk reduction, and number needed to treat. **Results:** PRT produced significantly greater improvements than PNE across eight of eleven outcomes, including anxiety, fear avoidance, pain catastrophizing, kinesiophobia, pain-related anxiety, self-efficacy for rehabilitation, and pain acceptance, with medium-to-large effect sizes (ηp^2^ = 0.088–0.316). Both groups improved in depressive symptoms and pain self-efficacy. Trait anger did not significantly change in either group. The number needed to treat for PRT was 2 for self-efficacy for rehabilitation and 3 for pain catastrophizing, kinesiophobia, and pain acceptance. **Conclusions:** PRT demonstrated greater effectiveness across eight psychological risk and resilience factors than PNE, warranting further investigation in larger trials.

## 1. Introduction

Chronic low back pain (CLBP) is one of the most prevalent and disabling musculoskeletal conditions worldwide [1], yet its complexity extends beyond structural pathology alone [2]. A growing body of evidence suggests that psychological factors are not merely secondary consequences of CLBP pain but key determinants of its onset, maintenance, and severity [3,4].

Vulnerability factors such as catastrophizing, fear of movement, anxiety, and depression have been consistently associated with greater pain intensity, functional limitation, and long-term disability in individuals with CLBP [5,6,7]. These pain-related psychological factors have been shown to constrain maximal physical performance and drive avoidance behavior, creating self-perpetuating cycles of deconditioning and disability that are difficult to interrupt through biomedical means alone [8,9]. Psychological factors also emerge as significant predictors of treatment outcomes [4], including those following surgical intervention, and are strongly associated with quality of life and the biopsychosocial determinants of chronicity [10,11]. Indeed, elevated psychological distress serves as a robust long-term predictor of chronicity, underscoring the prognostic significance of these factors well before pain becomes entrenched [3,12]. Understanding vulnerability factors provides insights into the risk factors known to influence CLBP [13]. However, addressing risk factors in isolation fails to account for the psychological resources that enable individuals to adapt to pain, pointing to the need for a framework that incorporates resilience alongside risk [14].

Resilience is a known predictor of pain-related outcomes [15]. New evidence suggests a salient role of resilience in pain adaptation and successful restoration of function [16]. Resilience is protective, buffering the effects of pain-related vulnerability factors [17]. Emerging resilience factors include hope [18], optimism [19], and zest [20]. More well-established resilience factors are pain acceptance [21,22] and self-efficacy [23,24]. When assessed and addressed together, risk and resilience factors provide an opportunity to address a broader multidimensional set of psychological factors related to pain [14,25]. Collectively, this evidence compels clinicians and researchers alike to position that the thorough assessment of psychological factors is not an adjunct to CLBP evaluation; rather, it is an indispensable component of comprehensive, patient-centered care. Considering the strong link between psychological factors and pain-related disability, interventions targeting these factors could enhance the quality of life of those with CLBP. These observations have catalyzed a fundamental reconceptualization of CLBP [26], with international clinical guidelines advocating for assessment and management strategies that systematically address psychological contributors alongside physical impairments [27], with implications for multidisciplinary pain care [28,29,30].

Pain Resilience Therapy (PRT) is a novel strengths-based approach recently developed to enhance pain recovery [31]. In a randomized controlled trial, our research group showed that PRT improved outcomes on pain intensity, pain resilience, and pain interference compared to Pain Neuroscience Education (PNE) in adults with CLBP [32]. However, important risk factors such as pain catastrophizing, kinesiophobia, and resilience factors such as self-efficacy and pain acceptance were not previously investigated as outcomes [7]. Understanding a broader profile of psychological risk and resilience factors is important for physical therapists working at the intersection of pain [33] and mental health [34,35].

Therefore, the objective of this secondary analysis was to evaluate the effectiveness of PRT compared to PNE on psychological risk and resilience factors in adults with chronic low back pain (CLBP). We hypothesized that PRT would produce greater improvements than PNE across a multidimensional profile of psychological risk and resilience factors in adults with CLBP. This analysis considered a multidimensional profile of pain-related distress and mental health factors. Findings from this study may inform evidence-based treatment selection and guide the development of targeted interventions aimed at these psychological factors, with the potential to improve the quality of life among individuals with CLBP.

## 2. Methods

### 2.1. Study Design and Procedures

This study presents a secondary analysis of data from a pragmatic, parallel, randomized controlled trial (RCT) comparing PRT with PNE in adults with CLBP. Briefly, 72 participants were randomized 1:1 to receive 8 sessions of PRT or PNE, delivered via telehealth twice weekly over 4 weeks, with outcomes assessed at baseline and at 90-day follow-up. The telehealth delivery and 90-day follow-up period were intentional, reflecting the pragmatic design of this trial and its emphasis on evaluating intervention effectiveness under real-world conditions [36]. The study was conducted in accordance with the Declaration of Helsinki, was approved by the Widener University Institutional Review Board (protocol #180-23), and was registered at ClinicalTrials.gov (#NCT06971809) in March 2025. The trial was conducted from May 2025 to March 2026. The trial was conducted and reported in accordance with the Consolidated Standards of Reporting Trials (CONSORT) 2025 statement [37]. Patients or members of the public were not involved in the design of this research.

This secondary analysis examined outcomes of PRT versus PNE on multidimensional psychological variables assessed by the Optimal Screening for Prediction of Referral and Outcome Yellow Flag (OSPRO-YF) Assessment Tool (Duke University, Durham, NC, USA) [38]. Data from all participants who completed the parent trial were included in this analysis. The OSPRO-YF was pre-specified as a secondary outcome measure in the original trial registration. The parent trial protocol and statistical analysis plan are available from the corresponding author upon reasonable request. The statistical approach for this secondary analysis followed the same ANCOVA framework as the parent trial, with the addition of post hoc treatment response analyses. After identifying statistically significant between-group differences, treatment response analyses of relative risk, absolute risk reduction, and number needed to treat were calculated to permit clinical interpretation of results.

### 2.2. Participants

**Figure 1:**
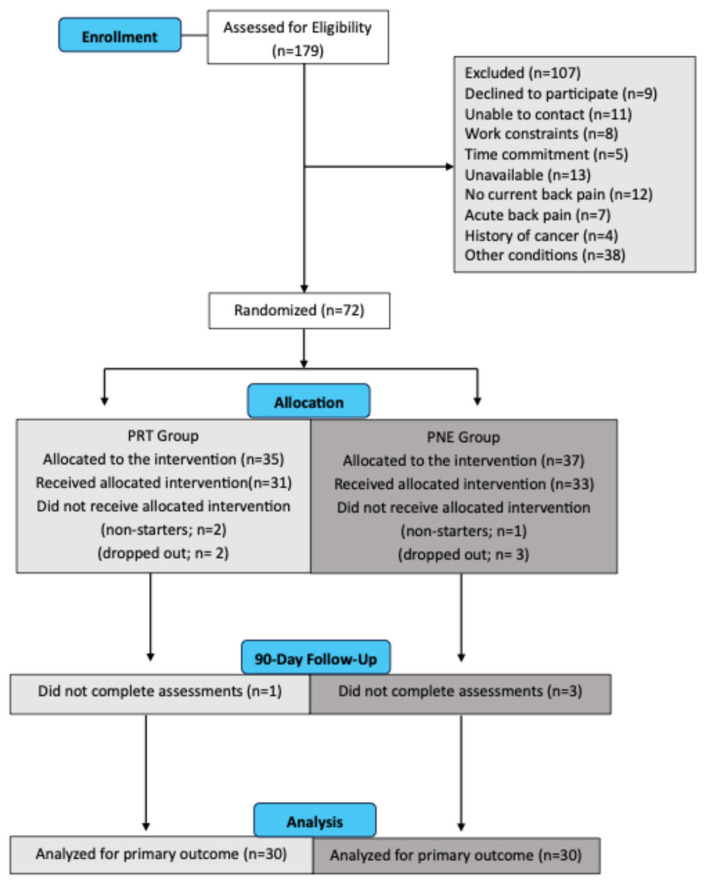
Participant flow chart.

Potential participants were recruited via social media and through an established listserv maintained by the Integrative Pain Science Institute. Participants were required to be at least 18 years of age, speak and understand English, and have experienced CLBP for at least 6 months. Additional eligibility criteria required participants to have sufficient proficiency in English to read study materials, understand consent, and complete outcome measures; have access to the internet and a computer equipped with audio, video, and Zoom capability; and be available to participate twice weekly for 4 consecutive weeks. Exclusion criteria included individuals experiencing acute pain due to a recent injury, a history of metastatic cancer, or pain less than six months. Participants unable to read or comprehend English were also excluded to ensure fidelity in intervention delivery and data collection. Figure 1 shows the flow of participants through the study.

### 2.3. Interventions

The PNE and PRT telehealth sessions were provided in a synchronous, online format. Sessions were held twice weekly over four weeks. All sessions were led by a single physical therapist (JT) with more than 25 years of experience implementing multimodal physical therapy interventions in clinical practice, education, and research settings [33]. A single therapist was employed to minimize between-therapist variability. A structured session checklist was developed for each PRT and PNE session to ensure the core therapeutic components were covered as intended and to provide a systematic record of protocol adherence throughout the intervention period.

#### 2.3.1. Pain Neuroscience Education

PNE is an educational approach aimed at reconceptualizing pain in patients with CLBP [39]. Metaphors and analogies are used to explain neurophysiological pain processes. Studies demonstrate PNE effectively reduces pain, disability, kinesiophobia, and catastrophizing in CLBP [40,41]. Intervention details are in Supplementary Table S1.

#### 2.3.2. Pain Resilience Therapy

PRT is a strengths-based approach grounded in evidence-based positive psychology constructs [42] and modern principles of exposure derived from inhibitory learning theory [43]. PRT focuses on identifying and leveraging individuals’ innate capacities, resilience, and resources rather than centering on deficits. Therapeutic targets include savoring, an active cognitive process of training attention and regulating emotion, and cognitive–affective positivity targeting how patients think and feel about their pain. This is primarily achieved via acceptance, which reduces the struggle against pain as something that must be eliminated before life can resume. Mindfulness builds present-moment awareness without reactive judgment. Courage supports engagement with feared activities despite discomfort. Optimism fosters a forward-looking orientation toward recovery and possibility. Memory rescripting is a process of transforming negative or threatening pain-related images into adaptive, empowering narratives. New learning is used to address pain-related beliefs. Behavioral perseverance to remain engaged in meaningful behaviors and rehabilitation tasks is discussed, even in the presence of discomfort, and a strengths-exploration component is used to identify and activate existing personal strengths as recovery resources. Over time, there is a shift from focusing on symptom reduction (e.g., session # 1, savoring) to building capacity (e.g., session #7, taking courageous action). Intervention details are in Supplementary Table S1. A complete intervention description using the TIDieR checklist [44] is in Supplementary Table S2.

### 2.4. Blinding

Participants were not informed of their group assignment or the name of the intervention they received. They were aware that two approaches were being studied but were not told which they had been allocated to, thereby maintaining participant concealment with respect to group assignment. Given the behavioral nature of PRT and PNE, the treating therapist (JT) was necessarily aware of the intervention being delivered. To minimize performance and detection bias, all outcome measures were self-reported by participants via an online survey platform with no access to prior results. One researcher (KP) conducted the data analysis to prevent clinician bias. The researcher responsible for statistical analysis was not blinded to group allocation.

### 2.5. Randomization

All participants provided informed consent before data collection. A computer-generated random number sequence (https://www.graphpad.com/quickcalcs/randomize2/) accessed on 1 June 2025 was used to allocate participants to groups. The allocation sequence was held exclusively by a central coordinator who was not involved in participant enrollment or intervention delivery. Group assignments were communicated to participants only after eligibility was confirmed and confirmation and informed consent was obtained, ensuring that the sequence remained concealed from enrolling personnel prior to assignment. The treating therapist (JT) was responsible for confirming participant eligibility and obtaining informed consent but did not have access to the allocation sequence. Group assignment was communicated to participants by the central coordinator, thereby maintaining separation between the enrollment and allocation functions.

### 2.6. Harms

Adverse events were defined as any new symptom or health event not present at baseline, spontaneously reported by the participant related to study participation. In-session fluctuations in pain intensity did not meet the criteria for adverse events. Any adverse events were recorded by the treating therapist.

### 2.7. Outcome Measures

Outcomes for this secondary analysis were assessed using the Optimal Screening for Prediction of Referral and Outcome Yellow Flag (OSPRO-YF) Assessment Tool, which was pre-specified in the parent trial protocol. Participants in both groups were assessed prior to beginning the intervention and again at 90 days post intervention.

The 10-item OSPRO-YF tool was used to measure pain-related vulnerability and resilience factors. It includes 2 domains related to risk (negative mood and negative/maladaptive coping) and 1 domain related to resilience (positive affect/coping). The Cronbach’s alpha coefficients for the three domains are 0.88, 0.94, and 0.94, respectively [45]. The OSPRO-YF is a valid and reliable multidimensional psychological measure for individuals with chronic pain and accurately estimates scores of 11 full-length psychological questionnaires [38]. The OSPRO-YF has a minimum of 81% accuracy to estimate scores on the Pain Self-Efficacy Questionnaire (PSEQ), Patient Health Questionnaire (PHQ-9), State-Trait Anxiety Inventory (STAI), State-Trait Anger Expression Inventory (STAXI), Fear-Avoidance Beliefs Questionnaire physical activity subscale (FABQ-PA), Fear-Avoidance Beliefs Questionnaire work subscale (FABQ-W), Pain Catastrophizing Scale (PCS), Tampa Scale of Kinesiophobia (TSK-11), Pain Anxiety Symptoms Scale (PASS-20), Self-Efficacy for Rehabilitation (SER), and the Chronic Pain Acceptance Questionnaire (CPAQ). See Table 1 for a description of the surveys for which the OSPRO-YF provides score estimates.

### 2.8. Sample Size Calculation

An a priori power analysis was conducted using G*Power v. 3.1.9.7 to determine the required sample size for the parent trial. Assuming a large effect size, power of 0.80, an alpha level of 0.05, and a one-tailed directional significance, the analysis indicated a total sample size of 56 participants (28 per group) was required for the primary outcomes. No interim analyses were planned or conducted for this secondary analysis. The sample size was determined by the parent trial design.

### 2.9. Data Analysis

Data were analyzed using SPSS v. 29 [56]. Demographic data were analyzed using descriptive statistics, including frequencies, means, and standard deviations, when appropriate. Means and standard deviations were calculated for the PNE and PRT group pre-test and 90-day post-test scores for all OSPRO-YF estimated measures. All 60 participants included in this secondary analysis provided complete OSPRO-YF data at both time points; therefore, no imputation of missing data was required.

Pre-specified between-groups comparison of post-test outcomes was conducted using analysis of covariance (ANCOVA), with baseline adjustment to provide statistical control for the pre-test scores. Prior to conducting the primary ANCOVA, group × baseline interactions were tested to evaluate the assumption of homogeneity of regression slopes necessary for ANCOVA analysis. Holm–Bonferroni corrections were applied separately to families of interaction and main effect tests. Interaction effects were evaluated first, followed by Holm–Bonferroni-adjusted main effects for those outcomes without significant interactions [57].

To provide context for the study results, post hoc relative risk, absolute risk reduction, and number needed to treat analyses were performed for all significant group × time interactions. Estimated outcomes for these variables were dichotomized as improved or not improved using the reported minimal detectable change (MDC) for each original measure, which represents a change beyond error [58]. Frequencies were calculated for these variables.

## 3. Results

### 3.1. Participant Characteristics

Participant recruitment consisted of 179 individuals assessed for eligibility; 107 did not meet the inclusion criteria. Seventy-two eligible individuals provided informed consent and were randomized: 35 to PRT and 37 to PNE. Sixty participants completed the eight-session intervention and the 90-day follow-up assessment and were included in this secondary analysis (30 per group). The analytical sample of 60 participants had a mean age of 51.7 years (SD 16.7) in the PNE group and 48.1 (12.9) in the PRT group. The majority of participants were women, married, college graduates, employed full-time, and were not using opioid pain medication. Participants were permitted to continue pre-existing medical treatments and medications throughout the trial. See Table 2 for percentages of demographic characteristics.

### 3.2. Outcomes

The Group × baseline interaction for the primary outcome PSEQ was statistically significant (*p* < 0.001). Additionally, the secondary outcome PHQ9 also demonstrated a significant interaction (*p* = 0.010) after Holm–Bonferroni correction. All other interaction effects were not statistically significant after correction.

For the PSEQ, the PRT group had higher scores at baseline, with a large standard deviation, indicating greater variability around the mean. At post-test, the PRT group mean decreased, with a smaller standard deviation, which may indicate regression towards the mean. For the PHQ-9, participants in the PRT group had lower scores at baseline, which may have impacted the lower scores at post-test (Table 3). After adjusting for baseline, main effects were found for all other variables, except for the STAXI, *F*(1, 57) = 2.240, *p* = 0.140, ηp^2^ = 0.038. The FABQ-W and FABQ-PA had a medium effect size (ηp^2^ = 0.088–0.112); all other effect sizes were large (Table 3).

### 3.3. Treatment Response Analysis

In order to place the significant main effects within the context of treatment response, post hoc relative risk, absolute risk reduction, and number needed to treat were calculated for the STAI, FABQ-PA, FABQ-W, PCS, PASS-20, TSK-11, SER, and CPAQ (Table 4).

No adverse events were reported among any of the 72 randomized participants (PRT n = 35; PNE n = 37) during the 4-week intervention period or at 90-day follow-up.

Participants receiving PRT were more likely to achieve meaningful improvement across all significant main effects compared to those receiving PNE. For the STAI, participants in the PRT group were 1.62 times more likely to improve, corresponding to an absolute difference of 17 percentage points. Treating six people with chronic pain with PRT would result in one additional person reducing their anxiety.

For the FABQ-PA, participants in the PRT group were 1.62 times more likely to improve, corresponding to an absolute difference of 17 percentage points. Treating six people with chronic pain with PRT would result in one additional person experiencing a reduction in fear avoidance of physical activity.

For the FABQ-W, participants in the PRT group were 2.25 times more likely to improve, corresponding to an absolute difference of 17 percentage points. Treating six people with chronic pain with PRT would result in one additional person experiencing a reduction in fear avoidance of work.

For the PCS, participants in the PRT group were 2.83 times more likely to experience reduced pain catastrophizing, corresponding to an absolute difference of 37 percentage points. Treating three people with chronic pain with PRT would result in one additional person reducing pain catastrophizing.

For the TSK-11, participants in the PRT group were 4.0 times more likely to experience reduced kinesiophobia, corresponding to an absolute difference of 40 percentage points. Treating three people with chronic pain with PRT would result in one additional person reducing kinesiophobia.

For the PASS-20, participants in the PRT group were 1.8 times more likely to experience reduced pain-related anxiety, corresponding to an absolute difference of 13 percentage points. Treating eight people with chronic pain with PRT would result in one additional person reducing pain-related anxiety.

For the SER, participants in the PRT group were 1.6 times more likely to improve, corresponding to an absolute difference of 30 percentage points. Treating two people with chronic pain with PRT would result in one additional person improving their self-efficacy for rehabilitation.

For the CPAQ, participants in the PRT group were 6.5 times more likely to experience improvement, corresponding to an absolute difference of 37 percentage points. Treating three people with chronic pain with PRT would result in one additional person improving in chronic pain acceptance. See Table 5 for all treatment responses.

## 4. Discussion

### 4.1. Interpretation of Findings

This secondary analysis examined the comparative effectiveness of PRT versus PNE on psychological risk and resilience factors in adults with CLBP. After controlling for baseline differences, PRT demonstrated superior outcomes across the majority of psychological domains assessed, including anxiety, fear avoidance, pain catastrophizing, kinesiophobia, pain anxiety, self-efficacy for rehabilitation, and chronic pain acceptance. These findings extend the primary trial results and provide insight into the psychological mechanisms through which PRT may confer clinical benefit.

### 4.2. Risk Factors: Negative Mood and Maladaptive Coping

PRT produced meaningfully greater reductions in pain catastrophizing, kinesiophobia, pain-related anxiety, and fear-avoidance beliefs compared to PNE, with large effect sizes across these constructs. Treatment response analyses underscore the clinical significance of these differences: participants receiving PRT were 2.83 times more likely to achieve a clinically meaningful reduction in pain catastrophizing and 4.0 times more likely to reduce kinesiophobia. The numbers needed to treat (NNT) values are 3 for pain catastrophizing and 3 for kinesiophobia, indicating high effectiveness.

PNE has demonstrated effectiveness in reducing pain catastrophizing and kinesiophobia in prior trials and systematic reviews with NNT ranging from 4 to 6 [40]. Therefore, it is noteworthy that PRT produced superior outcomes on these constructs compared with PNE and may reflect the additive value of PRT’s positive psychology and inhibitory learning framework. From the perspective of the fear-avoidance model, reductions in kinesiophobia and fear-avoidance beliefs are of particular clinical importance, as these constructs perpetuate avoidance behavior, pain intensity, and long-term disability [66]. PRT’s grounding in inhibitory learning, which emphasizes the creation of new safety-based associations rather than mere fear extinction, may be well suited to disrupt self-reinforcing cycles in CLBP.

Trait anxiety was also significantly reduced in the PRT group relative to PNE (ηp^2^ = 0.162; NNT ≈ 6), consistent with evidence that positive psychology interventions can regulate negative affective states. Trait anger did not differ significantly between groups, which may reflect the lower salience of anger-related constructs within PRT’s therapeutic framework, or may suggest that anger in chronic pain requires more targeted interventions and/or longer treatment to yield detectable change. Depression scores demonstrated a significant group-by-baseline interaction, and the baseline imbalance in PHQ-9 scores warrants caution in attributing differential change to treatment alone.

### 4.3. Resilience Factors: Positive Affect and Coping

The most clinically compelling findings concern the resilience outcomes. PRT produced large improvements in both self-efficacy for rehabilitation (ηp^2^ = 0.285) and chronic pain acceptance (ηp^2^ = 0.316). Participants in the PRT group were 6.5 times more likely to achieve a clinically meaningful improvement in pain acceptance and 1.6 times more likely to improve meaningfully in self-efficacy for rehabilitation. The NNT values are 3 for pain acceptance and 2 for self-efficacy in rehabilitation, indicating high effectiveness.

These findings are theoretically coherent with PRT’s strengths-based design. Self-efficacy is among the most robust predictors of rehabilitation engagement and long-term functional outcomes in CLBP [23,24]. Pain acceptance has been associated with superior pain outcomes and quality of life across multiple systematic reviews [21,22,67]. PNE, while effective at modifying pain beliefs, does not explicitly target pain acceptance or self-efficacy. PRT’s therapeutic components, including savoring, mindfulness, acceptance, cognitive–affective positivity, and strengths exploration, appear to provide distinct mechanisms for fostering these resilience capacities.

This pattern is consistent with recommendations to both reduce risk and build resilience [14]. The positive psychology literature demonstrates that resilience-focused interventions can simultaneously reduce negative affect and augment positive psychological resources [68]. The concurrent reduction of risk factors and enhancement of resilience factors observed in PRT aligns with contemporary evidence emphasizing that chronic pain outcomes are determined not only by the presence of risk but also by the degree to which protective resources buffer their effects [14,69].

### 4.4. Contextualizing PRT Within the Active Comparator

PNE is an established educational approach for CLBP [40,41,70] widely used by physical therapists [71]. In this study, PNE produced within-group improvements across some of the psychological domains assessed (anxiety, fear-avoidance beliefs, pain catastrophizing, kinesiophobia, pain-related anxiety), alongside modest gains in self-efficacy for rehabilitation and pain acceptance. These within-group changes are consistent with PNE’s established evidence base of small-to-moderate effect sizes for short-term outcomes [41]. However, when examined against minimal detectable change thresholds (Table 4), participants in the PNE group were substantially less likely to achieve clinically meaningful improvement across these domains relative to those receiving PRT. Scholars have emphasized the need to integrate PNE with behavioral interventions to optimize outcomes [72,73]. However, direct comparisons between PNE and active behavioral approaches remain extremely scarce in the literature [74]. Recent trials comparing PNE to ergonomics education [75] or examining PNE as an adjunct to interdisciplinary care have not demonstrated additional benefits [76]. The differential effectiveness favoring PRT therefore emerges in the context of comparison to an active behavioral treatment rather than an inert control [77]. Finally, PRT may be considered within a broader body of evidence relevant to the present findings. First, positive psychology interventions combined with pain education have been shown to reduce pain and enhance psychological resilience in adults with CLBP [78]. Second, multimodal physical therapist interventions produce greater preventive [79] and therapeutic effects [80] on pain, disability, and kinesiophobia than exercise or education alone.

### 4.5. Strengths and Limitations

This is the second study to investigate PRT, and its findings meaningfully strengthen the evidence base for this approach. Critically, this study measured a broad range of both psychological risk and resilience factors using a validated, multidimensional assessment tool. This dual-focus approach [14,33] is rarely reported in the pain literature and reflects the most current models for understanding chronic pain outcomes [33]. The use of an RCT with an active, clinically credible comparator rather than a waitlist or attention-only control substantially elevates the interpretive weight of the findings, ensuring that observed differences reflect genuine therapeutic specificity rather than nonspecific effects of care. The consistent superiority of PRT across eight of eleven psychological domains, with medium-to-large effect sizes, strengthens confidence in the breadth and specificity of its effectiveness. Furthermore, the inclusion of treatment response analyses of relative risk, absolute risk reduction, and number needed to treat moves beyond statistical significance to quantify clinically meaningful benefit, with participants in the PRT group up to 6.5 times more likely to achieve meaningful improvement on key outcomes. The successful delivery of both interventions via telehealth demonstrates the feasibility and accessibility of both PNE and PRT in real-world physical therapist practice, expanding their potential reach to populations who might otherwise face barriers to in-person care.

This study has several limitations. The OSPRO-YF provides estimated rather than directly administered scores. This may introduce measurement imprecision and potentially limit its sensitivity for detecting positive or negative change in psychological outcomes such as depression, anxiety, and anger [81]. The post hoc relative risk, absolute risk reduction, and number needed to treat analysis should be interpreted with caution, as the minimal detectable change values used are based on the original measures rather than the score estimate. Given the lack of validated MCIDs for OSPRO-YF estimated scores, as well as evidence that MCIDs may vary based on factors such as patient population, study conditions, and the choice of anchors in MCID calculations, MDCs were used for RR, ARR, and NNT calculations to determine improvement beyond error [58,82].

The significant group-by-baseline interaction for the PHQ-9 limits the interpretability of depressive symptom outcomes. The power analysis was performed for the parent trial, which may yield an underpowered study, risking a Type II error. The inclusion of effect sizes and confidence intervals provides context for the results, which should be considered exploratory. To address the risk of Type I error, Holm–Bonferroni corrections were applied. A single therapist delivered all sessions. True blinding was not achieved as the therapist was unblinded by necessity, the analyst was aware of group allocation, and the therapist’s dual role as PRT developer and interventionist introduces therapist allegiance and expectancy bias; however, both the PRT and PNE groups demonstrated improvement. The sample was predominantly white, female, and highly educated, and outcomes were assessed at only two time points, constraining generalizability and conclusions about durability. Although PRT demonstrated greater improvements than PNE across several psychological outcomes, these findings require confirmation in adequately powered trials using directly administered measures and clearly defined analysis populations. Future research should include dismantling studies and mediation analyses to identify PRT’s active mechanisms; extended follow-up to assess durability; and larger, more diverse samples to enhance generalizability.

## 5. Conclusions

This secondary analysis of an RCT provides evidence that PRT leads to significant improvements in psychological risk and resilience factors among adults with CLBP. These results provide an important empirical foundation supporting the effectiveness of PRT and its use among adults with CLBP.

## Figures and Tables

**Table 1:** Domain and surveys estimated by the OSPRO-YF.

Domain	Tool	Description
Negative Mood	PHQ-9	The 9-item tool measures depression. Higher scores indicate greater depressive symptoms [46].
	STAI	The 20-item trait subscale of the STAI measures personality tendencies towards anxiety. Higher scores indicate greater anxiety [47].
	STAXI	The 10-item trait subscale of the STAXI measures personality tendencies towards anger. Higher scores indicate greater levels of anger [48].
Negative/Maladaptive Coping	FABQ-PA	This 4-item subscale measures fear avoidance of physical activity due to chronic musculoskeletal pain [49]. Higher scores indicate greater levels of fear avoidance.
	FABQ-W	This 7-item subscale measures fear avoidance of work due to chronic musculoskeletal pain. Higher scores indicate greater levels of fear avoidance [49].
	PCS	The 13-item survey measures the tendency towards an exaggerated negative outlook towards anticipated or actual musculoskeletal pain. Higher scores indicate greater levels of pain catastrophizing [50].
	TSK-11	The 11-item survey measures the degree of fear of movement, injury and reinjury. Higher scores indicate greater levels of fear [51].
	PASS-20	The 20-item survey measures the degree of pain-related anxiety in people with pain diagnoses. Higher scores indicate higher levels of pain anxiety [52].
Positive Affect/Coping	PSEQ	The 10-item tool measures self-efficacy related to pain coping. Higher scores indicate greater pain self-efficacy [53].
	SER	The 12-item tool assesses self-efficacy for engaging in rehabilitation. Higher scores indicate greater self-efficacy [54].
	CPAQ	The 20-item tool assesses pain coping behavior. Higher scores indicate greater coping and pain acceptance [55].

Abbreviations: OSPRO-YF: Optimal Screening for Prediction of Referral and Outcome Yellow Flag; PHQ-9: Patient Health Questionnaire-9; STAI: State-Trait Anxiety Inventory; STAXI: State-Trait Anger Expression Inventory; FABQ-PA: Fear-Avoidance Beliefs Questionnaire physical activity subscale; FABQ-W: Fear-Avoidance Beliefs Questionnaire work subscale; PCS: Pain Catastrophizing Scale; TSK-11: Tampa Scale of Kinesiophobia; PASS-20: Pain Anxiety Symptoms Scale; PSEQ: Pain Self-Efficacy Questionnaire; SER: Self-Efficacy for Rehabilitation; CPAQ: Chronic Pain Acceptance Questionnaire.

**Table 2:** Participant characteristics of study completers for each treatment group.

Participant Characteristic		Frequency	
		PNE	PRT
Gender Identity	Man	8	13
	Woman	22	16
	Not listed	N/A	1
Race/Ethnicity	Asian/Pacific Islander	2	2
	Black/Non-Hispanic	1	N/A
	Hispanic	4	2
	White/Non-Hispanic	22	23
	Other	1	3
Marital Status	Married	13	20
	Not Married	17	10
Education	College Graduate	21	28
	Some College Education	9	1
	High School	N/A	1
Employment Status	Unemployed	14	5
	Part-Time (5–30 h/week)	9	7
	Full-Time (>30h/week)	7	18
Current Opioid Use	Yes	6	5
	No	24	25

Abbreviations: PNE: pain neuroscience education, PRT: pain resilience therapy.

**Table 3:** Outcome measures: post-test adjusted means and confidence intervals.

	PNE		PRT	
Outcome	90-Day Post-Test Mean	95% CI	90-Day Post-Test Mean	95% CI
PSEQ	32.8	29.8, 35.8	39.3	36.3, 42.4
PHQ-9	11.2	10.1, 12.2	7.1	6.1, 8.2
STAI *	45.1	43.2, 47.0	40.7	38.8, 42.6
STAXI	20.4	18.8, 22.1	18.7	17.0, 20.3
FABQ-PA ^†^	14.7	13.8, 15.7	13.0	12.0. 13.9
FABQ-W ^‡^	22.2	19.3, 25.0	17.5	14.6, 20.3
PCS ^§^	23.9	21.4, 26.4	18.0	15.5, 20.5
TSK-11 ^¶^	26.8	25.5, 28.1	22.3	21.0, 23.6
PASS-20 ^#^	39.3	35.9, 42.8	31.5	28.1, 34.9
SER **	80.3	75.4, 85.2	97.0,	92.1, 101.9
CPAQ ^††^	59.5	56.0, 63.0	72.1	68.6, 75.6

Note: All *p*-values presented reflect Holm–Bonferroni corrections. * Main effect of group for the STAI, F(1, 57) = 11.0, *p* = 0.009, ηp^2^ = 0.162, 95% CI [0.026, 0.327]. ^†^ Main effect of group for the FABQ-PA, F(1, 57) = 7.204, *p* = 0.020, ηp^2^ = 0.112, 95% CI [0.007, 0.272]. ^‡^ Main effect of group for the FABQ-W, F(1, 57) = 5.480, *p* = 0.023, ηp^2^ = 0.088, 95% CI [0, 0.072]. ^§^ Main effect of group for the PCS, F(1, 57) = 11.187, *p* = 0.009, ηp^2^ = 0.164, 95% CI [0.027, 0.330]. ^¶^ Main effect of group for the TSK-11, F(1, 57) = 22.810, *p* < 0.009, ηp^2^ = 0.286, 95% CI [0.104, 0.447]. ^#^ Main effect of group for the PASS-20, F(1, 57) = 10.133, *p* = 0.009, ηp^2^ = 0.151, 95% CI [0.021, 0.316]. ** Main effect of group for the SER, F(1, 57) = 22.722, *p* < 0.009, ηp^2^ = 0.285, 95% CI [0.103, 0.446]. ^††^ Main effect of group for the CPAQ, F(1, 57) = 26.371, *p* < 0.009, ηp^2^ = 0.316, 95% CI [0.128, 0.383]. Abbreviations: PNE: Pain Neuroscience Education; PRT: Pain Resilience Therapy; CI: Confidence Interval; PHQ-9: Patient Health Questionnaire-9; STAI: State-Trait Anxiety Inventory; STAXI: State-Trait Anger Expression Inventory; FABQ-PA: Fear-Avoidance Beliefs Questionnaire physical activity subscale; FABQ-W: Fear-Avoidance Beliefs Questionnaire work subscale; PCS: Pain Catastrophizing Scale; TSK-11: Tampa Scale of Kinesiophobia; PASS-20: Pain Anxiety Symptoms Scale; PSEQ: Pain Self-Efficacy Questionnaire; SER: Self-Efficacy for Rehabilitation.

**Table 4:** Frequency of improvement by the MDC for significant group × time interaction variables.

Variable	Change (Points)	Yes		No	
		PNE	PRT	PNE	PRT
STAI	8 [59]	8	13	22	17
FABQ-PA	9.4 [60]	8	13	22	17
FABQ-W	12.7 [60]	4	9	26	21
PCS	8 [61]	6	17	24	13
TSK-11	5.6 [62]	4	16	26	14
PASS-20	15.6 [63]	5	9	25	21
SER	2.48 [64]	15	24	15	6
CPAQ	22 [65]	2	13	28	17

Abbreviations: MDC: Minimal Detectable Change; PNE: Pain Neuroscience Education; PRT: Pain Resilience Therapy; STAI: State-Trait Anxiety Inventory; FABQ-PA: Fear-Avoidance Beliefs Questionnaire physical activity subscale; FABQ-W: Fear-Avoidance Beliefs Questionnaire work subscale; PCS: Pain Catastrophizing Scale; TSK-11: Tampa Scale of Kinesiophobia; PASS-20: Pain Anxiety Symptoms Scale; SER: Self-Efficacy for Rehabilitation.

**Table 5:** Relative risk and absolute risk reduction estimates and number needed to treat for treatment response.

	Relative Risk	Absolute Risk Reduction	Number Needed to Treat
STAI	1.62	0.17	6
FABQ-PA	1.62	0.17	6
FABQ-W	2.25	0.17	6
PCS	2.83	0.37	3
TSK-11	4.0	0.40	3
PASS-20	1.80	0.13	8
SER	1.60	0.30	2
CPAQ	6.50	0.37	3

Abbreviations: STAI: State-Trait Anxiety Inventory; FABQ-PA: Fear-Avoidance Beliefs Questionnaire physical activity subscale; FABQ-W: Fear-Avoidance Beliefs Questionnaire work subscale; PCS: Pain Catastrophizing Scale; TSK-11: Tampa Scale of Kinesiophobia; PASS-20: Pain Anxiety Symptoms Scale; SER: Self-Efficacy for Rehabilitation.

## Data Availability

The deidentified data presented in this study are available on request from the corresponding author due to privacy and ethical reasons.

## Supplementary Materials

The following supporting information can be downloaded at: https://www.mdpi.com/article/10.3390/healthcare14131940/s1, Table S1: Therapeutic Elements of PNE and PRT; Table S2: TIDieR Checklist Table for Pain Resilience Therapy. Refs. [83,84,85,86,87,88,89,90] are cited in the Supplementary Material file.

## Abbreviations

The following abbreviations are used in this manuscript.

ANCOVA	Analysis of Covariance
CLBP	Chronic Low Back Pain
CONSORT	Consolidated Standards of Reporting Trials
CPAQ	Chronic Pain Acceptance Questionnaire
FABQ-PA	Fear-Avoidance Beliefs Questionnaire (Physical Activity)
FABQ-W	Fear-Avoidance Beliefs Questionnaire (Work)
MDC	Minimal Detectable Change
NNT	Number Needed to Treat
OSPRO-YF	Optimal Screening for Prediction of Referral and Outcome Yellow Flag
PASS-20	Pain Anxiety Symptoms Scale (20-item)
PCS	Pain Catastrophizing Scale
PHQ-9	Patient Health Questionnaire (9-item)
PNE	Pain Neuroscience Education
PRT	Pain Resilience Therapy
PSEQ	Pain Self-Efficacy Questionnaire
SER	Self-Efficacy for Rehabilitation
SPSS	Statistical Package for the Social Sciences
STAI	State-Trait Anxiety Inventory
STAXI	State-Trait Anger Expression Inventory
TSK-11	Tampa Scale of Kinesiophobia (11-item)
TIDieR	Template for Intervention Description and Replication
