# Supplementary material for: Pain Resilience Therapy vs. Pain Neuroscience Education for Adults with Chronic Low Back Pain: Secondary Analysis of Data from a Randomized Controlled Trial of Risk and Resilience Factors
Source: Healthcare (Basel). 2026 Jul 1;14(13):1940. doi: 10.3390/healthcare14131940 (PMC13362344; doi:10.3390/healthcare14131940)
Supplement: Supplementary file 1 [file healthcare-14-01940-s001.zip › healthcare-4326615-supplementary.pdf]

**Supplementary Table S1.** Therapeutic Elements of PNE and PRT.

| Session | PNE | PRT |
|---|---|---|
| 1 | Pain Knowledge<br>Pain neuroscience education introduced. Improving knowledge of how pain works in promoting recovery emphasized Current knowledge and understanding of pain-related topics explored to create a baseline. | Learning to Savor the Experience of Safety in the Body<br>Education on the human pain system, how pain reduces positive feelings, and positive feelings reduce pain. Participants practice cultivating the ability to savor and sustain the embodied experience of bodily safety (40). After the initial practice they generalize the experience while transitioning from sitting, standing, walking, and returning. |
| 2 | Sensitive Nerves<br>Education on the nervous system as the body's alarm system and the role of pain as a danger warning signal. Pain explained using metaphors to promote deep learning. | Harnessing Positive Future Thinking<br>The focus is on developing the skill of positive future thinking. Participants learn to challenge pain myths, use memory and imagery to envision hopeful outcomes, and build optimism about recovery (41). |
| 3 | Nerve Sensor<br>Education on the concept of neuroplasticity or the ability of the brain to adapt, and how factors such as temperature, stress, movement, immunity and blood flow affect pain. Instruction included how over-active pain may occur based on what the brain thinks is needed for survival. | Building Mindbody Resilience<br>This session emphasizes interconnected mindbody resilience through the practice of self-awareness and self-regulation to address interconnected thoughts, emotions, and bodily sensations. Participants willingly practice being in the presence of pain while it emerges noticing perceptual characteristics of mass, temperature, motion and cohesion (42,43). The exercise is practiced while transitioning from sitting, standing, walking, and returning. |
| 4 | Nosy Neighbors (spreading pain):<br><br>Education about spreading pain symptoms, and that feeling pain in nearby areas of the body does not indicate definite injury. Pain explained using metaphors to promote deep learning. | Pain Memory Rescripting<br>Participants are introduced to memory rescripting, a process of transforming negative or threatening pain-related images that intensify pain into new, preferred, adaptive or empowering alternatives. Positive sleep behaviors are introduced here as salient factors for both pain memory reconsolidation and pain reduction (44). |
| 5 | Calming Nerves<br>Education about the body's natural mechanisms and strategies to increase the brain's production of chemicals, which decrease pain, such as aerobic exercise and improved pain knowledge. The concepts of pacing, graded exposure, 'sore but safe', and 'hurt does not equal harm' discussed. Sleep hygiene and diaphragmatic breathing topics introduced to help calm the nervous system and reduce stress. | Moving with Zest 1<br>The skill focus is on pairing rhythmic movement to the beat of a metronome (45) while progressively increasing physical demands of spinal movement in various planes, including velocity and range of motion. Participants first demonstrate their ability to maintain rhythm through finger tapping, then select an individualized tempo they perceive as achievable (e.g., 55 beats per minute). Initial practice involves synchronizing simple spinal flexion to the metronome (5 counts down, 5 counts up), paired with positive instructional and motivational self-talk (46). |
| 6 | Pain and the Brain<br>Education about the concept of pain as an output of the brain, including the nervous system processing of noxious stimuli versus pain, inhibition and facilitation, and threat value using metaphors to promote deep learning. | Moving with Zest 2<br>Building on the prior week, participants continue practicing rhythmic movement increasing the tempo (e.g., increasing from 55 beats per minute to 75 beats per minute) with the use of a metronome (45), while progressively increasing physical demands of spinal |

| | | |
|---|---|---|
| | | movement in various planes, including velocity and range of motion. |
| 7 | Brain's Pain Map<br>Education about the pain map via functional MRI examples, and how various areas of the brain are involved in pain experience. These areas have an alternative primary focus, which is disturbed when the brain is producing pain (memory, focus, concentration, emotion, fine motor control, temperature), causing potential patient issues/ struggles. | Taking Courageous Action<br>This session centers on persistence in the face of pain through tasks of physical bravery. Participants are asked to categorize behaviors into non-courageous (surrendering to threat, giving up), coping (meeting basic needs, getting by), and courageous (pushing beyond the norm, completing a physical task that would surprise them or create joy.) Participants learn to take deliberate risks, tolerate discomfort, and act with courage in service of valued-based actions (47). |
| 8 | Body, Inc.'s CEO (central sensitization)<br>Education about nerve sensitivity caused by central sensitization, driven by both biological and psychosocial factors. The brain as CEO of the body metaphor incorporated. | Optimizing Strengths<br>The final session reviews learned skills and integrates them with participants' existing individual strengths exploration. Attention is given to how protective factors allow a person to be resilient in the face of challenges. A facilitated discussion on protective factors includes strengths such as activity, persistence, a positive outlook, self-compassion, support, experiencing positive emotions, having courage, knowledge/insight, and various positive lifestyle behaviors (48). Participants practice identifying these strengths to foster recovery, maintain engagement in life to continue "getting back out there." |

Abbreviations
PNE: Pain Neuroscience Education
PRT: Pain Resilience Therapy


1. DuPont, C.; Hunt, C.A.; Hanley, A.W.; Garland, E.L.; Finan, P.H. Does a Savoring Meditation Reduce Unpleasant Bodily Sen-sations or Increase Pleasant Bodily Sensations in Individuals With Rheumatoid Arthritis Relative to a Sham Meditation? J. Pain 2023, 24, 57. https://doi.org/10.1016/j.jpain.2023.02.171.
2. Basten-Günther, J.; Peters, M.; Lautenbacher, S. Optimism and the Experience of Pain: A Systematic Review. Behav. Med. 2019, 45, 323–339. https://doi.org/10.1080/08964289.2018.1517242. PMID: 30570408.
3. Cayoun, B.; Simmons, A.; Shires, A. Immediate and lasting chronic pain reduction following a brief self-implemented mind-fulness-based interoceptive exposure task: A pilot study. Mindfulness 2020, 11, 112–124. https://doi.org/10.1007/s12671-017-0823-x.
4. Paschali, M.; Lazaridou, A.; Sadora, J.; Papianou, L.; Garland, E.L.; Zgierska, A.E.; Edwards, R.R. Mindfulness-based Interventions for Chronic Low Back Pain: A Systematic Review and Meta-analysis. Clin. J. Pain 2024, 40, 105. https://doi.org/10.1097/AJP.0000000000001173.
5. Arntz, A. Imagery Rescripting: An update of the treatment protocol. Behav. Res. Ther. 2025, 195, 104913. https://doi.org/10.1016/j.brat.2025.104913.
6. Lee, H.K.; Kim, H.J.; Kim, S.B.; Kang, N. A Review and Meta-Analysis of Interactive Metronome Training: Positive Effects for Motor Functioning. Percept. Mot. Skills 2022, 129, 1614–1634. https://doi.org/10.1177/00315125221110403.

7. Chowkase, A.A.; Parra-Martínez, F.A.; Ghahremani, M.; Bernstein, Z.; Finora, G.; Sternberg, R.J. Dual-process model of courage. Front. Psychol. 2024, 15, 1376195. https://doi.org/10.3389/fpsyg.2024.1376195.
8. Braunwalder, C.; Müller, R.; Glisic, M.; Fekete, C. Are Positive Psychology Interventions Efficacious in Chronic Pain Treatment? A Systematic Review and Meta-Analysis of Randomized Controlled Trials. Pain Med. 2022, 23, 122–136. https://doi.org/10.1093/pm/pnab247.

**Supplementary Table S2.** TIDieR Checklist Table for Pain Resilience Therapy.

| TIDieR Item | Pain Resilience Therapy Description |
|---|---|
| 1. Brief name | Pain Resilience Therapy (PRT). |
| 2. Why (rationale, theory, goal) | Resilience and strengths-based interventions target positive valence mechanisms; aims to reduce pain intensity/interference and increase pain resilience. |
| 3. What—Materials | Zoom for Healthcare; patient handouts & practice logs; exercises for savoring safety, positive imagery, self-regulation; sleep-health checklist; metronome (app/web) for movement sessions, clinician presentation materials for psychoeducation. |
| 4. What—Procedures | 8 tele-physical therapy sessions (twice weekly × 4 weeks) following Table 1: 1) savoring bodily safety; 2) positive future thinking; 3) mind–body self-regulation; 4) pain-memory rescripting + sleep behaviors; 5–6) "moving with zest" (metronome-entrained graded movement + positive self-talk); 7) courageous action; 8) strengths optimization & maintenance planning. Between-session practices assigned to consolidate learning. |
| 5. Who provided | One licensed physical therapist (JT) with specific training in PRT, trauma-informed and psychologically-informed care. |
| 6. How (modes of delivery) | Synchronous tele-physical therapy via Zoom for Healthcare. Hybrid in-person option was offered but not utilized in this cohort. |
| 7. Where | Participant home/private setting with secure telehealth; therapist in clinical/office environment. Infrastructure: device with camera/audio, stable internet, metronome access via smart phone. |
| 8. When & how much | 8 sessions, 60 minutes each, delivered twice weekly over 4 weeks. PRT group was instructed to practice all exercises 3x/day to retrain neuroplasticity. |
| 9. Tailoring | Content individualized to pain resilience, movement tolerance, and values/strengths. For movement sessions, metronome tempo, range of motion, and task complexity were titrated to maintain challenge with safety and perceived control. Imagery/rescripting targets were personalized. |
| 10. Modifications | None reported during the cohort; the eight-session structure, telehealth mode, and core content remained unchanged. |
| 11. How well—Planned | Standardized session plan and documentation of core elements (psychoeducation, experiential practices, metronome use/tempo, values-based action plans). Single-provider delivery minimized variability. No independent fidelity audits were planned. |
| 12. How well—Actual | All completers received the planned 8-session telehealth program. |

Abbreviation:
TIDieR: Template for Intervention Description and Replication
PRT: Pain Resilience Therapy
